# Synthesis and crystal structure of *catena*-poly[[bis­(nitrato-κ^2^*O*,*O*′)strontium(II)]-di-μ-l-histidine-κ^3^*O*,*O*′:*O*;κ^2^*O*:*O*′]

**DOI:** 10.1107/S2056989025004694

**Published:** 2025-06-10

**Authors:** Sathish Marimuthu, Thangavelu Balakrishnan, M. Judith Percino, Perumal Venkatesan

**Affiliations:** a Crystal Growth Laboratory PG and Research Department of Physics Thanthai Periyar Government Arts and Science College (Autonomous and affiliated to Bharathidasan University, Tiruchirappalli) Tiruchirappalli-620 023 Tamil Nadu India; b Unidad de Polímeros y Electrónica Orgánica, Instituto de Ciencias, Benemérita, Universidad Autónoma de Puebla, Val3-Ecocampus Valsequillo, Independencia O2 Sur 50, San Pedro Zacachimalpa, 72960, Puebla, Mexico; c Department of Chemistry Srimad Andavan Arts and Science College (Autonomous and affiliated to Bharathidasan University, Tiruchirappalli) Tiruchirappalli-620 005 Tamil Nadu India; University of Aberdeen, United Kingdom

**Keywords:** crystal structure, coordination polymer, l-histidine, strontium

## Abstract

The title mono-periodic coordination polymer, [Sr(NO_3_)_2_(C_6_H_9_N_3_O_2_)_2_]_*n*_, was synthesized from l-histidine and strontium nitrate. Crystallizing in the monoclinic space group *C*2, the structure features an Sr^2+^ cation (site symmetry 2) coordinated by ten oxygen atoms from zwitterionic l-histidine ligands and nitrate anions, forming a distorted deca­hedral geometry with Sr—O bond lengths ranging from 2.645 (4) to 2.863 (4) Å. The bridging l-histidine mol­ecules generate a polymeric chain extending along the [010] direction. The structure is consolidated by N—H⋯O, N—H⋯N and weak C—H⋯O hydrogen bonds, creating layers lying parallel to the *bc* plane.

## Abstract 2

The title mono-periodic coordination polymer features ten-coordinate Sr^2+^ ions, zwitterionic l-histidine ligands and nitrate anions.

## 1. Chemical context

Coordination polymers and metal–organic frameworks are hybrid inorganic–organic materials characterized by extended crystal structures formed through coordination bonds between metal ions or metal-containing bridged clusters and multifunctional organic ligands. These structures can form chains, layers or three-dimensional networks, making them highly versatile materials (*e.g*., Tăbăcaru *et al.*, 2018 ▸; Jiao *et al.*, 2019 ▸; Pettinari *et al.*, 2016 ▸). Over the past few decades, these phases have garnered significant attention from chemists, material scientists, and physicists, both in academia and industry, due to their remarkable structural diversity and wide-ranging applications in fields such as sensors (Wu *et al.*, 2020 ▸; Wang, 2020 ▸), catalysis (Wu & Zhao, 2017 ▸; Zhu *et al.*, 2017 ▸), photonics (Dhakshinamoorthy *et al.*, 2016 ▸), gas storage and separation (Farrusseng, 2011 ▸), electronics (Baumann *et al.*, 2019 ▸) and other applications.

Al-Terkawi *et al.* (2017 ▸) reported the synthesis and structural analysis of strontium coordination polymers containing deprotonated tetra­fluoro­phthalic and phthalic acids. They synthesized two strontium-based di­carboxyl­ate systems by a mechanochemical method and determined their structures from powder X-ray diffraction data. Dynamic vapor sorption tests showed no significant difference in adsorption behavior between dehydrated and hydrated samples. Zhang *et al.* (2014 ▸) synthesized and characterized a strontium(II) coordination polymer with pyridine-2,6-di­carboxyl­ate anions, sulfate ions and water mol­ecules. The crystal structure of this coordination polymer is consolidated by hydrogen bonding and π–π stacking inter­actions. Fei *et al.* (2005 ▸) described the synthesis and crystal structure of a hydrated di-periodic strontium–imidazolium carboxyl­ate coordination polymer. The crystal structure reveals that the polymeric sheets are separated by near-planar water sheets. The water mol­ecules form edge-sharing hexa­gons linked by O—H⋯O hydrogen bonds and establish hydrogen bonds with the imidazolium ions, while not inter­acting with the strontium cations.
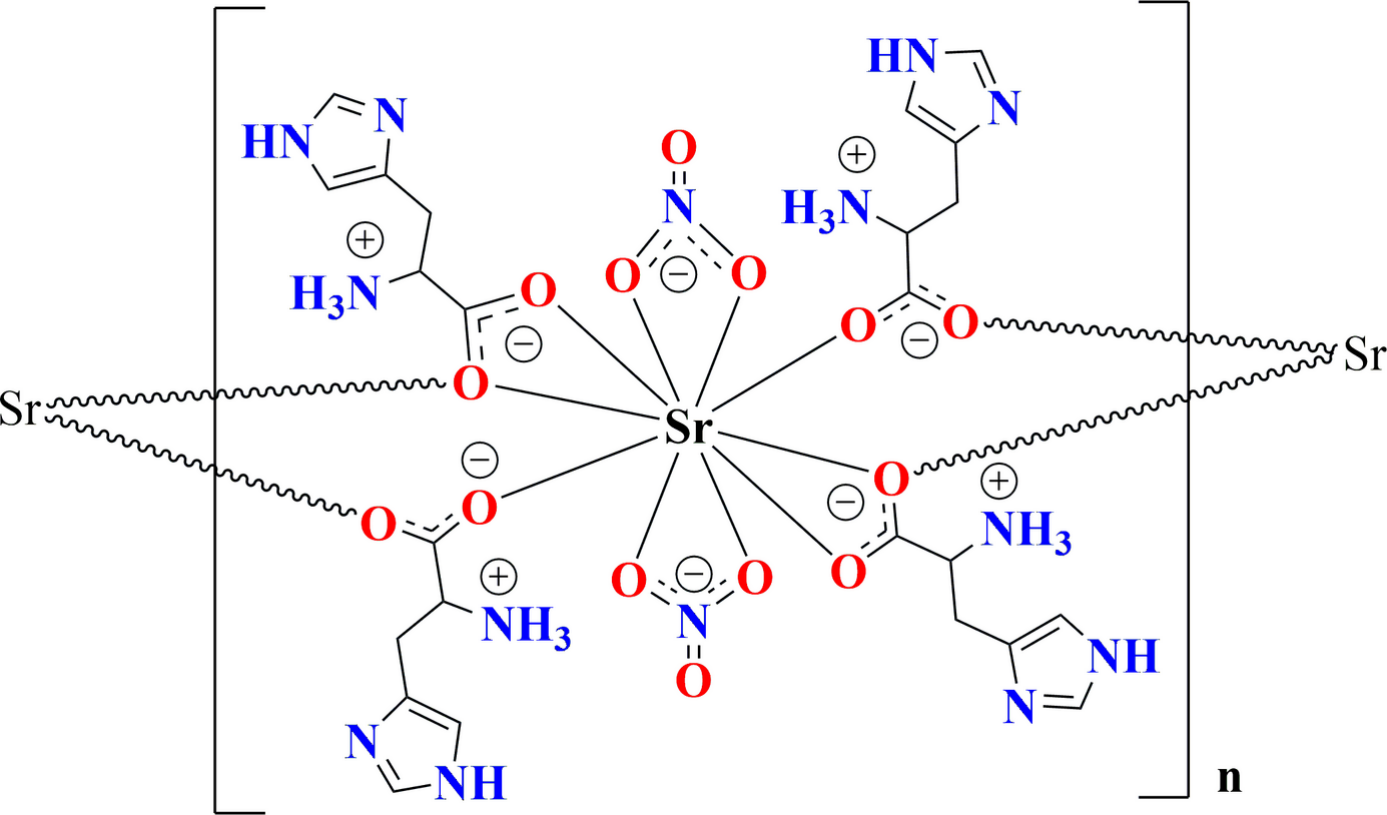


l-histidine-based crystal structures have attracted significant attention in materials science due to their diverse properties and wide-ranging applications (Thangavel *et al.*, 2022 ▸; Pereira *et al.*, 2023 ▸; Li *et al.*, 2022 ▸). The ability of l-histidine (C_6_H_9_N_3_O_2_) to coordinate with metals makes it an effective reagent for preparing metal–organic frameworks used in catalysis and adsorption. Additionally, these structures exhibit nonlinear optical properties, as well as piezoelectric and ferroelectric characteristics. Building on these earlier findings, and as part of our ongoing research into l-histidine-based metal–organic frameworks, we present here the synthesis and crystal structure of the title mono-periodic coordination polymer, [Sr(NO_3_)_2_(C_6_H_9_N_3_O_2_)_2_]_*n*_ (**I**), formed from the reaction of l-histidine and strontium nitrate in aqueous solution.

## 2. Structural commentary

**Figure 1:**
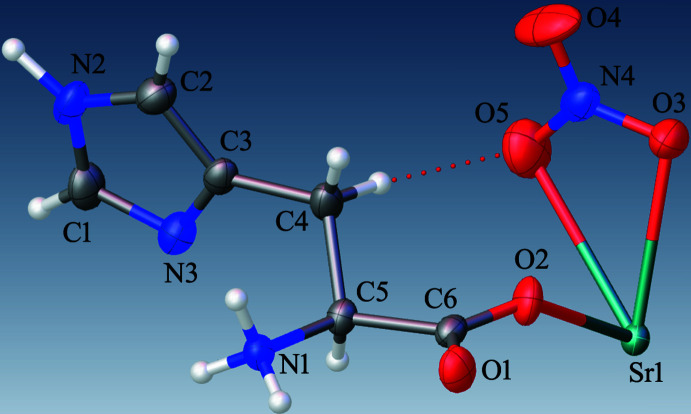
The asymmetric unit of (**I**) showing 50% displacement ellipsoids.

**Figure 2:**
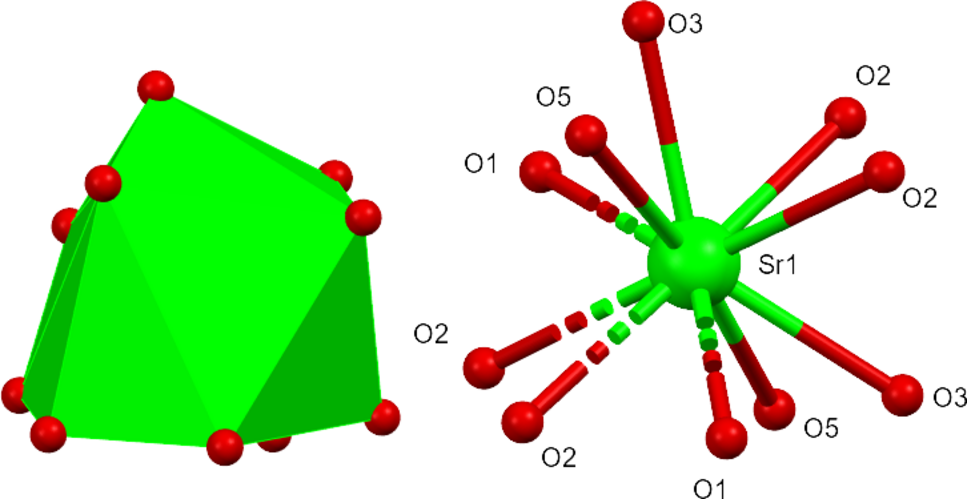
A view of the coordination environment surrounding the strontium atom in (**I**).

Compound (**I**) crystallizes in the monoclinic space group *C*2. The asymmetric unit consists of one zwitterionic l-histidine mol­ecule, one nitrate anion and one Sr^2+^ cation (Fig. 1 ▸). The bond lengths (Table 1 ▸) around the carboxyl­ate group of the l-histidine mol­ecule indicate deprotonation, *i.e*., carrying a negative charge. The amine group of the l-histidine is protonated, resulting in its zwitterionic form in the crystal structure. The Sr^2+^ ion lies on a crystallographic twofold axis and is coordinated by ten oxygen atoms (Fig. 2 ▸). Among them, four originate from two l-histidine mol­ecules that simultaneously chelate the Sr^2+^ cation *via* atoms O1 and O2, while two others come from two different bridging l-histidine mol­ecules (*via* O2). The remaining four oxygen atoms belong to two chelating nitrate anions, which act as *O*,*O*-bidentate ligands. As a result, the Sr^2+^ cation adopts a distorted deca­hedral geometry. The Sr—O bond lengths range from 2.645 (4) to 2.863 (4) Å while the O—Sr—O angles vary between 45.60 (9) and 146.16 (10)°. These values are comparable to previously reported data (Parsekar *et al.*, 2022 ▸; Natarajan *et al.*, 2011 ▸; Arularasan *et al.*, 2013 ▸). The bond lengths and bond angles in the l-histidine mol­ecule are consistent with earlier reported crystal structures (Gokul Raj *et al.*, 2006 ▸; Raghavalu *et al.*, 2007 ▸; Muralidharan *et al.*, 2013 ▸) : key torsion angles are presented in Table 1 ▸. The expected *S* configuration of C5 is confirmed by the refinement. The bridging l-histidine mol­ecules connect neighboring metal ions into a polymeric chain propagating along [010] *via* atom O2, resulting in a Sr⋯Sr separation of 4.6574 (5) Å for adjacent metal ions (Fig. 2 ▸), *i.e*., the *b* cell dimension.

## 3. Supra­molecular features

**Figure 3:**
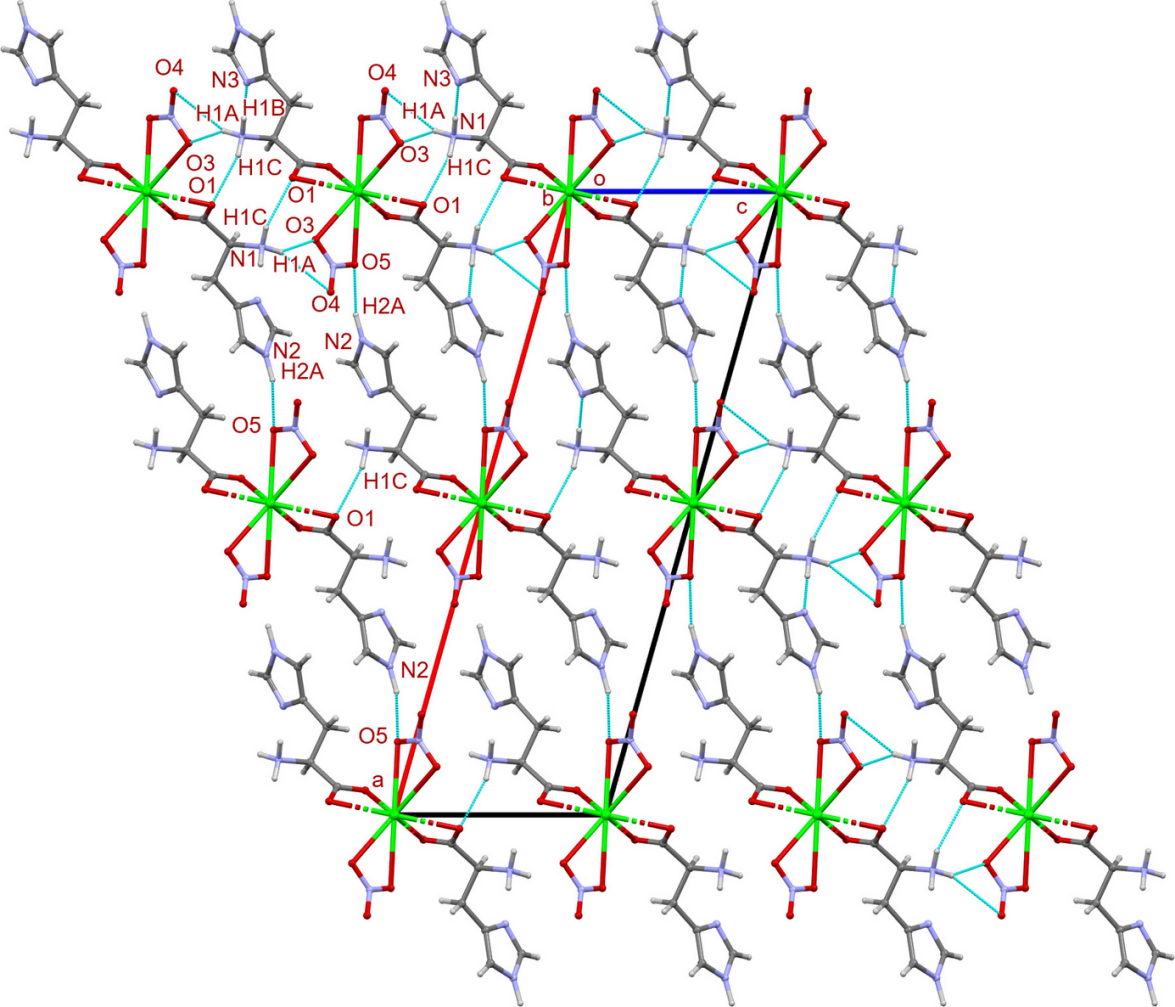
A view along the *b*-axis showing the packing of (**I**) with hydrogen bonds indicated by blue dashed lines.

In the extended structure, each polymeric chain is inter­connected with neighboring chains *via* hydrogen bonds, including N2—H2*A*⋯O5 and N1—H1*B*⋯O1 (Table 2 ▸). The structure is consolidated by additional hydrogen-bonding inter­actions, including N1—H1*B*⋯N3, N1—H1*A*⋯O3, N1—H1*A*⋯O4, and N1—H1*C*⋯O1. Finally, the N1—H1*A*⋯O3 inter­action results in the formation of a three-dimensional supra­molecular architecture (Table 2 ▸, Fig. 3 ▸).

## 4. Database survey

A search of the Cambridge Structural Database (CSD, Version 5.45, update of June 2024; Groom *et al.*, 2016 ▸) using Conquest (Bruno *et al.*, 2002 ▸) for a zwitterionic l-histidine mol­ecule gave 87 hits, ten of which involved coordination to metal atoms, *viz*.: Zn [CSD refcode: DOBBEC01 (Mekhatria *et al.*, 2011 ▸), KEKWIH (Chen & Bu 2006 ▸), NADWIA (Dong *et al.*, 2010 ▸), and YAHLOJ (Fan *et al.*, 2005 ▸)], Rh (EYEWUD; Jalilehvand *et al.*, 2021 ▸), Cd [HADXOD (Seo & Ok 2021 ▸), KITCIC (Sihem *et al.*, 2019 ▸), KITCIC01 (Mohamedi *et al.*, 2019 ▸), and KITCIC02 (Seo & Ok 2021 ▸)], Ag (TIGHEY; Mirolo *et al.*, 2013 ▸). In contrast, a search for the neutral non-zwitterionic l-histidine mol­ecule gave 28 hits, six of which displayed metal coordination: Pt [FARYUS (Baidina *et al.*, 1990 ▸), and KUWQEZ (Ye *et al.*, 2009 ▸)], Hg (HISHGC; Adams *et al.*, 1970 ▸), Ir (SETMOT; Krämer *et al.*, 1990 ▸), Pd (VIWSUP01; Caubet *et al.*, 1992 ▸), and V (WEGFIX01; Czernuszewicz *et al.*, 1994 ▸). The coordination modes varied across these structures: mono *O*-coordination through the carboxyl group was observed in complexes with Zn (DOBBEC01), Cd (HADXOD, KITCIC, KITCIC 01, KITCIC 02), Zn (KEKWIH), and Hg (HISHGC). A bidentate *O*,*O*-coordination mode was seen in LIKREE (Arularasan *et al.*, 2013 ▸). Coordination *via* N atoms either the amino group, the imidazole-ring nitro­gen atom, or both, was evident in Pt (FARYUS, and KUWQEZ), Ir (SETMOT), Pd (VIWSUP01).

## 5. Synthesis and crystallization

In a 250 ml beaker, l-histidine (1.552 g) and strontium nitrate (2.12 g) were taken in equimolar amounts and dissolved in deionized water (30 ml) at room temperature. The mixture was stirred thoroughly for 4 h using a magnetic stirrer and then filtered. The beaker was placed in an undisturbed area to allow the mother solution to slowly evaporate. After 12 days, colorless single crystals of (**I**) with a well-formed triangular shape were harvested with dimensions of up to 0.9 × 0.4 × 0.3 mm.

## 6. Refinement

Crystal data, data collection and structure refinement details are summarized in Table 3 ▸. The N-bound H atoms were located in a difference-Fourier map and refined with isotropic displacement parameters. All C-bound H atoms were included in calculated positions and treated as riding atoms with C—H = 0.93–0.98 Å and *U*_iso_(H) = 1.2*U*_eq_(C). The crystal studied was refined as a two-component inversion twin.

## Supplementary Material

Crystal structure: contains datablock(s) I, global. DOI: 10.1107/S2056989025004694/hb8141sup1.cif

Structure factors: contains datablock(s) I. DOI: 10.1107/S2056989025004694/hb8141Isup2.hkl

CCDC reference: 2453805

Additional supporting information:  crystallographic information; 3D view; checkCIF report

## Figures and Tables

**Table 1:** Selected geometric parameters (Å, °)

Sr1—O2	2.645 (4)	Sr1—O2^ii^	2.863 (4)
Sr1—O3	2.674 (2)	C6—O2	1.245 (4)
Sr1—O1^i^	2.681 (2)	C6—O1	1.257 (4)
Sr1—O5	2.824 (3)		
			
Sr1—O2—Sr1^iii^	115.42 (6)		
			
C3—C4—C5—N1	−55.2 (4)	N1—C5—C6—O2	−177.5 (2)
C3—C4—C5—C6	−177.7 (3)	C4—C5—C6—O2	−54.8 (4)

Symmetry codes: (i) 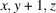; (ii) 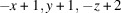; (iii) 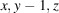.

**Table 2:** Hydrogen-bond geometry (Å, °)

*D*—H⋯*A*	*D*—H	H⋯*A*	*D*⋯*A*	*D*—H⋯*A*
N1—H1*B*⋯N3^iii^	0.90 (2)	1.92 (2)	2.818 (3)	179 (3)
N1—H1*C*⋯O3^iv^	0.85 (2)	2.99 (4)	3.387 (4)	111 (3)
N1—H1*C*⋯O1^v^	0.85 (2)	2.24 (2)	3.039 (3)	156 (3)
N2—H2*A*⋯O4^vi^	1.00 (5)	2.25 (5)	2.854 (3)	118 (4)
N2—H2*A*⋯O5^vii^	1.00 (5)	2.28 (5)	3.180 (4)	148 (4)
C4—H4*B*⋯O5	0.97	2.54	3.507 (5)	175
C5—H5⋯O1^i^	0.98	2.55	3.373 (4)	142

Symmetry codes: (i) 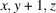; (iii) 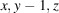; (iv) 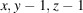; (v) 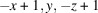; (vi) 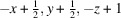; (vii) 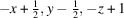.

**Table 3:** Experimental details

Crystal data	
Chemical formula	[Sr(NO_3_)_2_(C_6_H_9_N_3_O_2_)_2_]
*M*_r_	521.96
Crystal system, space group	Monoclinic, *C*2
Temperature (K)	298
*a*, *b*, *c* (Å)	24.9533 (7), 4.6575 (1), 8.1543 (2)
β (°)	105.695 (1)
*V* (Å^3^)	912.36 (4)
*Z*	2
Radiation type	Mo *K*α
μ (mm^−1^)	3.03
Crystal size (mm)	0.20 × 0.11 × 0.02

Data collection	
Diffractometer	Bruker D8 VENTURE diffractometer with PHOTON II detector
Absorption correction	Multi-scan (*SADABS*; Krause *et al.*, 2015 ▸)
*T*_min_, *T*_max_	0.593, 0.799
No. of measured, independent and observed [*I* > 2σ(*I*)] reflections	9589, 1859, 1859
*R*_int_	0.027
(sin θ/λ)_max_ (Å^−1^)	0.641

Refinement	
*R*[*F*^2^ > 2σ(*F*^2^)], *wR*(*F*^2^), *S*	0.016, 0.045, 1.13
No. of reflections	1859
No. of parameters	158
No. of restraints	7
H-atom treatment	H atoms treated by a mixture of independent and constrained refinement
Δρ_max_, Δρ_min_ (e Å^−3^)	0.36, −0.19
Absolute structure	Refined as an inversion twin.
Absolute structure parameter	0.037 (8)

Computer programs: *APEX4* and *SAINT* (Bruker, 2021 ▸), *SHELXT2014/5* (Sheldrick, 2015*a* ▸), *SHELXL2018/3* (Sheldrick, 2015*b* ▸), *ORTEP-3 for Windows* (Farrugia, 2012 ▸) and *Mercury* (Macrae *et al.*, 2020 ▸).

## supplementary crystallographic information

### catena-Poly[[bis(nitrato-κ2O,O')strontium(II)]-di-µ-L-histidine-κ3O,O':O;κ2O:O'] Crystal data

**Table:**

[Sr(NO_3_)_2_(C_6_H_9_N_3_O_2_)_2_]	*F*(000) = 528
*M**_r_* = 521.96	*D*_x_ = 1.900 Mg m^−^^3^
Monoclinic, *C*2	Mo *K*α radiation, λ = 0.71073 Å
*a* = 24.9533 (7) Å	Cell parameters from 9983 reflections
*b* = 4.6575 (1) Å	θ = 3.4–27.1°
*c* = 8.1543 (2) Å	µ = 3.03 mm^−^^1^
β = 105.695 (1)°	*T* = 298 K
*V* = 912.36 (4) Å^3^	Plate, colourless
*Z* = 2	0.20 × 0.11 × 0.02 mm

### catena-Poly[[bis(nitrato-κ2O,O')strontium(II)]-di-µ-L-histidine-κ3O,O':O;κ2O:O'] Data collection

**Table:**

Bruker D8 VENTURE diffractometer with PHOTON II detector	1859 independent reflections
Radiation source: fine-focus sealed tube	1859 reflections with *I* > 2σ(*I*)
Graphite monochromator	*R*_int_ = 0.027
ω and φ scan	θ_max_ = 27.1°, θ_min_ = 3.4°
Absorption correction: multi-scan (SADABS; Krause *et al.*, 2015)	*h* = −31→31
*T*_min_ = 0.593, *T*_max_ = 0.799	*k* = −5→5
9589 measured reflections	*l* = −10→10

### catena-Poly[[bis(nitrato-κ2O,O')strontium(II)]-di-µ-L-histidine-κ3O,O':O;κ2O:O'] Refinement

**Table:**

Refinement on *F*^2^	Hydrogen site location: mixed
Least-squares matrix: full	H atoms treated by a mixture of independent and constrained refinement
*R*[*F*^2^ > 2σ(*F*^2^)] = 0.016	*w* = 1/[σ^2^(*F*_o_^2^) + (0.0202*P*)^2^ + 0.2576*P*] where *P* = (*F*_o_^2^ + 2*F*_c_^2^)/3
*wR*(*F*^2^) = 0.045	(Δ/σ)_max_ = 0.001
*S* = 1.13	Δρ_max_ = 0.36 e Å^−^^3^
1859 reflections	Δρ_min_ = −0.19 e Å^−^^3^
158 parameters	Absolute structure: Refined as an inversion twin.
7 restraints	Absolute structure parameter: 0.037 (8)
Primary atom site location: dual	

### catena-Poly[[bis(nitrato-κ2O,O')strontium(II)]-di-µ-L-histidine-κ3O,O':O;κ2O:O'] Special details

**Table:**

Geometry. All esds (except the esd in the dihedral angle between two l.s. planes) are estimated using the full covariance matrix. The cell esds are taken into account individually in the estimation of esds in distances, angles and torsion angles; correlations between esds in cell parameters are only used when they are defined by crystal symmetry. An approximate (isotropic) treatment of cell esds is used for estimating esds involving l.s. planes.
Refinement. Refined as a 2-component inversion twin.

### catena-Poly[[bis(nitrato-κ2O,O')strontium(II)]-di-µ-L-histidine-κ3O,O':O;κ2O:O'] Fractional atomic coordinates and isotropic or equivalent isotropic displacement parameters (Å^2^)

**Table:**

	*x*	*y*	*z*	*U*_iso_*/*U*_eq_	
Sr1	0.500000	0.90071 (5)	1.000000	0.01832 (9)	
C1	0.27741 (10)	0.8961 (15)	0.2136 (3)	0.0323 (5)	
H1	0.271782	1.046398	0.135723	0.039*	
C2	0.26207 (12)	0.5153 (7)	0.3481 (4)	0.0297 (6)	
H2	0.245095	0.357124	0.382380	0.036*	
C3	0.31540 (11)	0.6064 (6)	0.4157 (3)	0.0234 (5)	
C4	0.35847 (11)	0.4845 (7)	0.5642 (3)	0.0288 (7)	
H4A	0.345129	0.301837	0.594645	0.035*	
H4B	0.362698	0.612670	0.660601	0.035*	
C5	0.41568 (10)	0.4382 (8)	0.5331 (3)	0.0206 (6)	
H5	0.430841	0.625543	0.513984	0.025*	
C6	0.45482 (10)	0.3017 (6)	0.6917 (3)	0.0210 (5)	
O1	0.47792 (9)	0.0682 (5)	0.6740 (3)	0.0305 (5)	
O2	0.46051 (8)	0.4284 (9)	0.8299 (2)	0.0312 (5)	
O3	0.42251 (9)	0.6821 (6)	1.1355 (3)	0.0404 (6)	
O4	0.33890 (11)	0.5834 (8)	0.9953 (4)	0.0652 (9)	
O5	0.38281 (11)	0.9385 (10)	0.9198 (3)	0.0528 (9)	
N1	0.41140 (9)	0.2554 (5)	0.3799 (3)	0.0221 (4)	
N2	0.23858 (10)	0.7020 (6)	0.2203 (3)	0.0312 (5)	
N3	0.32485 (9)	0.8489 (5)	0.3311 (3)	0.0287 (7)	
N4	0.38007 (10)	0.7320 (6)	1.0161 (3)	0.0291 (5)	
H1A	0.4028 (12)	0.357 (7)	0.286 (3)	0.040 (10)*	
H1C	0.4414 (10)	0.165 (8)	0.386 (5)	0.043 (11)*	
H1B	0.3835 (11)	0.127 (6)	0.364 (4)	0.035 (10)*	
H2A	0.198 (2)	0.692 (12)	0.154 (6)	0.070 (15)*	

### catena-Poly[[bis(nitrato-κ2O,O')strontium(II)]-di-µ-L-histidine-κ3O,O':O;κ2O:O'] Atomic displacement parameters (Å^2^)

**Table:**

	*U*^11^	*U*^22^	*U*^33^	*U*^12^	*U*^13^	*U*^23^
Sr1	0.01760 (13)	0.02030 (14)	0.01563 (13)	0.000	0.00204 (9)	0.000
C1	0.0333 (12)	0.0333 (13)	0.0270 (11)	0.012 (2)	0.0027 (9)	0.006 (2)
C2	0.0239 (12)	0.0333 (13)	0.0314 (14)	0.0004 (11)	0.0064 (11)	−0.0006 (12)
C3	0.0196 (12)	0.0270 (12)	0.0225 (12)	0.0061 (10)	0.0040 (10)	−0.0031 (10)
C4	0.0234 (12)	0.042 (2)	0.0199 (11)	0.0091 (10)	0.0041 (10)	0.0014 (10)
C5	0.0199 (9)	0.0204 (19)	0.0189 (10)	0.0010 (11)	0.0011 (8)	0.0008 (11)
C6	0.0164 (11)	0.0236 (11)	0.0214 (12)	−0.0021 (8)	0.0020 (9)	0.0037 (9)
O1	0.0322 (10)	0.0312 (12)	0.0257 (10)	0.0112 (9)	0.0037 (8)	0.0065 (8)
O2	0.0321 (8)	0.0347 (14)	0.0203 (8)	−0.0028 (12)	−0.0041 (6)	−0.0038 (12)
O3	0.0265 (10)	0.0620 (16)	0.0309 (11)	−0.0031 (10)	0.0049 (9)	0.0114 (11)
O4	0.0360 (13)	0.087 (2)	0.077 (2)	−0.0342 (15)	0.0238 (14)	−0.0464 (18)
O5	0.0569 (14)	0.054 (2)	0.0441 (12)	0.0125 (17)	0.0086 (10)	0.0157 (17)
N1	0.0214 (10)	0.0264 (12)	0.0174 (10)	0.0018 (9)	0.0034 (8)	0.0017 (9)
N2	0.0204 (11)	0.0402 (14)	0.0280 (12)	0.0057 (10)	−0.0018 (9)	−0.0051 (11)
N3	0.0263 (10)	0.028 (2)	0.0284 (11)	0.0007 (9)	0.0024 (8)	−0.0024 (9)
N4	0.0232 (11)	0.0348 (15)	0.0283 (12)	0.0002 (10)	0.0050 (10)	−0.0090 (11)

### catena-Poly[[bis(nitrato-κ2O,O')strontium(II)]-di-µ-L-histidine-κ3O,O':O;κ2O:O'] Geometric parameters (Å, º)

**Table:**

Sr1—O2^i^	2.645 (4)	C3—C4	1.497 (4)
Sr1—O2	2.645 (4)	C4—C5	1.531 (3)
Sr1—O3^i^	2.674 (2)	C4—H4A	0.9700
Sr1—O3	2.674 (2)	C4—H4B	0.9700
Sr1—O1^ii^	2.681 (2)	C5—N1	1.492 (4)
Sr1—O1^iii^	2.681 (2)	C5—C6	1.532 (3)
Sr1—O5	2.824 (3)	C5—H5	0.9800
Sr1—O5^i^	2.824 (3)	C6—O2	1.245 (4)
Sr1—O2^iii^	2.863 (4)	C6—O1	1.257 (4)
Sr1—O2^ii^	2.863 (4)	O3—N4	1.251 (3)
C1—N3	1.325 (3)	O4—N4	1.212 (4)
C1—N2	1.337 (6)	O5—N4	1.256 (5)
C1—H1	0.9300	N1—H1A	0.878 (19)
C2—N2	1.362 (4)	N1—H1C	0.85 (2)
C2—C3	1.363 (4)	N1—H1B	0.90 (2)
C2—H2	0.9300	N2—H2A	1.00 (5)
C3—N3	1.376 (4)		
			
O2^i^—Sr1—O2	67.44 (12)	N2—C1—H1	124.0
O2^i^—Sr1—O3^i^	72.05 (7)	N2—C2—C3	106.4 (3)
O2—Sr1—O3^i^	71.04 (7)	N2—C2—H2	126.8
O2^i^—Sr1—O3	71.04 (7)	C3—C2—H2	126.8
O2—Sr1—O3	72.05 (7)	C2—C3—N3	109.5 (2)
O3^i^—Sr1—O3	135.25 (13)	C2—C3—C4	128.2 (3)
O2^i^—Sr1—O1^ii^	135.21 (7)	N3—C3—C4	122.3 (2)
O2—Sr1—O1^ii^	76.97 (8)	C3—C4—C5	114.6 (2)
O3^i^—Sr1—O1^ii^	71.26 (7)	C3—C4—H4A	108.6
O3—Sr1—O1^ii^	122.88 (6)	C5—C4—H4A	108.6
O2^i^—Sr1—O1^iii^	76.97 (8)	C3—C4—H4B	108.6
O2—Sr1—O1^iii^	135.21 (7)	C5—C4—H4B	108.6
O3^i^—Sr1—O1^iii^	122.88 (6)	H4A—C4—H4B	107.6
O3—Sr1—O1^iii^	71.26 (7)	N1—C5—C4	111.1 (2)
O1^ii^—Sr1—O1^iii^	146.16 (10)	N1—C5—C6	110.8 (3)
O2^i^—Sr1—O5	112.88 (10)	C4—C5—C6	109.2 (2)
O2—Sr1—O5	73.43 (11)	N1—C5—H5	108.6
O3^i^—Sr1—O5	138.34 (8)	C4—C5—H5	108.6
O3—Sr1—O5	45.60 (9)	C6—C5—H5	108.6
O1^ii^—Sr1—O5	80.21 (7)	O2—C6—O1	124.7 (3)
O1^iii^—Sr1—O5	97.68 (8)	O2—C6—C5	117.3 (3)
O2^i^—Sr1—O5^i^	73.43 (10)	O1—C6—C5	118.0 (2)
O2—Sr1—O5^i^	112.88 (10)	O2—C6—Sr1^iv^	67.8 (2)
O3^i^—Sr1—O5^i^	45.60 (9)	O1—C6—Sr1^iv^	59.51 (14)
O3—Sr1—O5^i^	138.34 (8)	C5—C6—Sr1^iv^	161.20 (19)
O1^ii^—Sr1—O5^i^	97.68 (8)	C6—O1—Sr1^iv^	96.65 (16)
O1^iii^—Sr1—O5^i^	80.21 (7)	C6—O2—Sr1	143.5 (2)
O5—Sr1—O5^i^	172.85 (19)	C6—O2—Sr1^iv^	88.4 (2)
O2^i^—Sr1—O2^iii^	115.42 (6)	Sr1—O2—Sr1^iv^	115.42 (6)
O2—Sr1—O2^iii^	177.14 (9)	N4—O3—Sr1	99.34 (17)
O3^i^—Sr1—O2^iii^	109.54 (7)	N4—O5—Sr1	92.04 (19)
O3—Sr1—O2^iii^	108.60 (7)	C5—N1—H1A	112 (3)
O1^ii^—Sr1—O2^iii^	100.50 (7)	C5—N1—H1C	112 (3)
O1^iii^—Sr1—O2^iii^	47.01 (7)	H1A—N1—H1C	109 (3)
O5—Sr1—O2^iii^	104.94 (10)	C5—N1—H1B	112 (2)
O5^i^—Sr1—O2^iii^	68.60 (10)	H1A—N1—H1B	104 (2)
O2^i^—Sr1—O2^ii^	177.14 (10)	H1C—N1—H1B	108 (3)
O2—Sr1—O2^ii^	115.42 (6)	C1—N2—C2	107.2 (2)
O3^i^—Sr1—O2^ii^	108.61 (7)	C1—N2—H2A	130 (3)
O3—Sr1—O2^ii^	109.54 (7)	C2—N2—H2A	123 (3)
O1^ii^—Sr1—O2^ii^	47.01 (7)	C1—N3—C3	104.9 (3)
O1^iii^—Sr1—O2^ii^	100.50 (7)	O4—N4—O3	120.7 (3)
O5—Sr1—O2^ii^	68.60 (10)	O4—N4—O5	122.5 (3)
O5^i^—Sr1—O2^ii^	104.94 (10)	O3—N4—O5	116.8 (3)
O2^iii^—Sr1—O2^ii^	61.72 (10)	O4—N4—Sr1	157.1 (2)
N3—C1—N2	112.0 (4)	O3—N4—Sr1	57.43 (14)
N3—C1—H1	124.0	O5—N4—Sr1	64.34 (16)
			
N2—C2—C3—N3	0.4 (3)	O1—C6—O2—Sr1	114.7 (4)
N2—C2—C3—C4	177.2 (3)	C5—C6—O2—Sr1	−66.8 (4)
C2—C3—C4—C5	132.6 (3)	Sr1^iv^—C6—O2—Sr1	133.1 (3)
N3—C3—C4—C5	−51.0 (4)	O1—C6—O2—Sr1^iv^	−18.4 (3)
C3—C4—C5—N1	−55.2 (4)	C5—C6—O2—Sr1^iv^	160.1 (2)
C3—C4—C5—C6	−177.7 (3)	N3—C1—N2—C2	−0.7 (4)
N1—C5—C6—O2	−177.5 (2)	C3—C2—N2—C1	0.2 (4)
C4—C5—C6—O2	−54.8 (4)	N2—C1—N3—C3	0.9 (4)
N1—C5—C6—O1	1.1 (3)	C2—C3—N3—C1	−0.7 (3)
C4—C5—C6—O1	123.8 (3)	C4—C3—N3—C1	−177.8 (3)
N1—C5—C6—Sr1^iv^	−76.2 (6)	Sr1—O3—N4—O4	153.1 (2)
C4—C5—C6—Sr1^iv^	46.5 (7)	Sr1—O3—N4—O5	−26.0 (3)
O2—C6—O1—Sr1^iv^	19.8 (3)	Sr1—O5—N4—O4	−154.9 (3)
C5—C6—O1—Sr1^iv^	−158.6 (2)	Sr1—O5—N4—O3	24.2 (3)

Symmetry codes: (i) −*x*+1, *y*, −*z*+2; (ii) *x*, *y*+1, *z*; (iii) −*x*+1, *y*+1, −*z*+2; (iv) *x*, *y*−1, *z*.

### catena-Poly[[bis(nitrato-κ2O,O')strontium(II)]-di-µ-L-histidine-κ3O,O':O;κ2O:O'] Hydrogen-bond geometry (Å, º)

**Table:**

*D*—H···*A*	*D*—H	H···*A*	*D*···*A*	*D*—H···*A*
N1—H1*B*···N3^iv^	0.90 (2)	1.92 (2)	2.818 (3)	179 (3)
N1—H1*C*···O3^v^	0.85 (2)	2.99 (4)	3.387 (4)	111 (3)
N1—H1*C*···O1^vi^	0.85 (2)	2.24 (2)	3.039 (3)	156 (3)
N2—H2*A*···O4^vii^	1.00 (5)	2.25 (5)	2.854 (3)	118 (4)
N2—H2*A*···O5^viii^	1.00 (5)	2.28 (5)	3.180 (4)	148 (4)
C4—H4*B*···O5	0.97	2.54	3.507 (5)	175
C5—H5···O1^ii^	0.98	2.55	3.373 (4)	142

Symmetry codes: (ii) *x*, *y*+1, *z*; (iv) *x*, *y*−1, *z*; (v) *x*, *y*−1, *z*−1; (vi) −*x*+1, *y*, −*z*+1; (vii) −*x*+1/2, *y*+1/2, −*z*+1; (viii) −*x*+1/2, *y*−1/2, −*z*+1.
